# Supplementary material for: A mechanistic theory for aquatic food chain length
Source: Nat Commun. 2017 Dec 11;8:2028. doi: 10.1038/s41467-017-02157-0 (PMC5725575; doi:10.1038/s41467-017-02157-0)
Supplement: Supplementary file 1 — Supplementary Information [file 41467_2017_2157_MOESM1_ESM.pdf]

Supplementary Note 1
Effect of variation in food web structure and functional response form on theory results

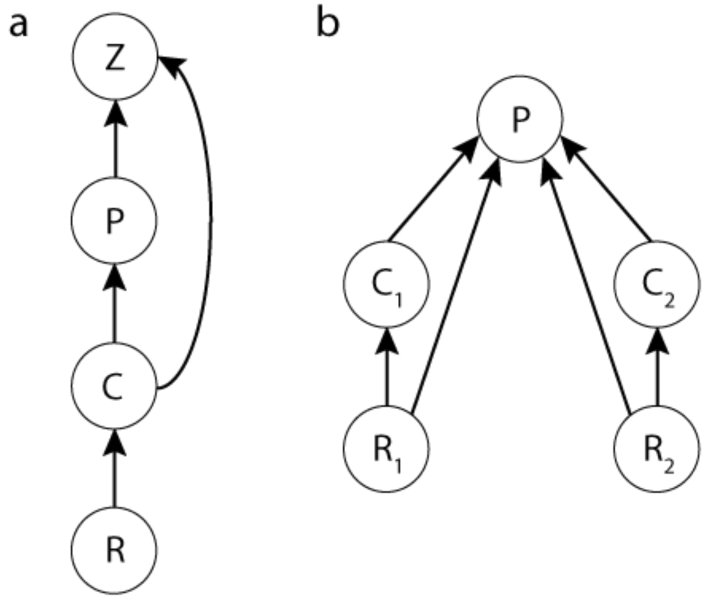

Supplementary Figure 1. Food web structures evaluated in sections (a) and (b).

(a) Food chain with four trophic levels (Supplementary Fig. 1a):

$$dR/dt = rR(1\text{-}R/K) - a_{CR}\,CR$$

$$dC/dt = e_{CR}\ a_{CR}\,CR - a_{PC}\,PC - a_{ZC}\,ZC - m_C\,C \qquad (S1)$$

$$dP/dt = e_{PC}\ a_{PC}\,PC - a_{ZP}\,ZP - m_P\,P$$

$$dZ/dt = e_{ZP}\ a_{ZP}\,ZP + e_{ZC}\ a_{ZC}\,ZC - m_Z\,Z$$

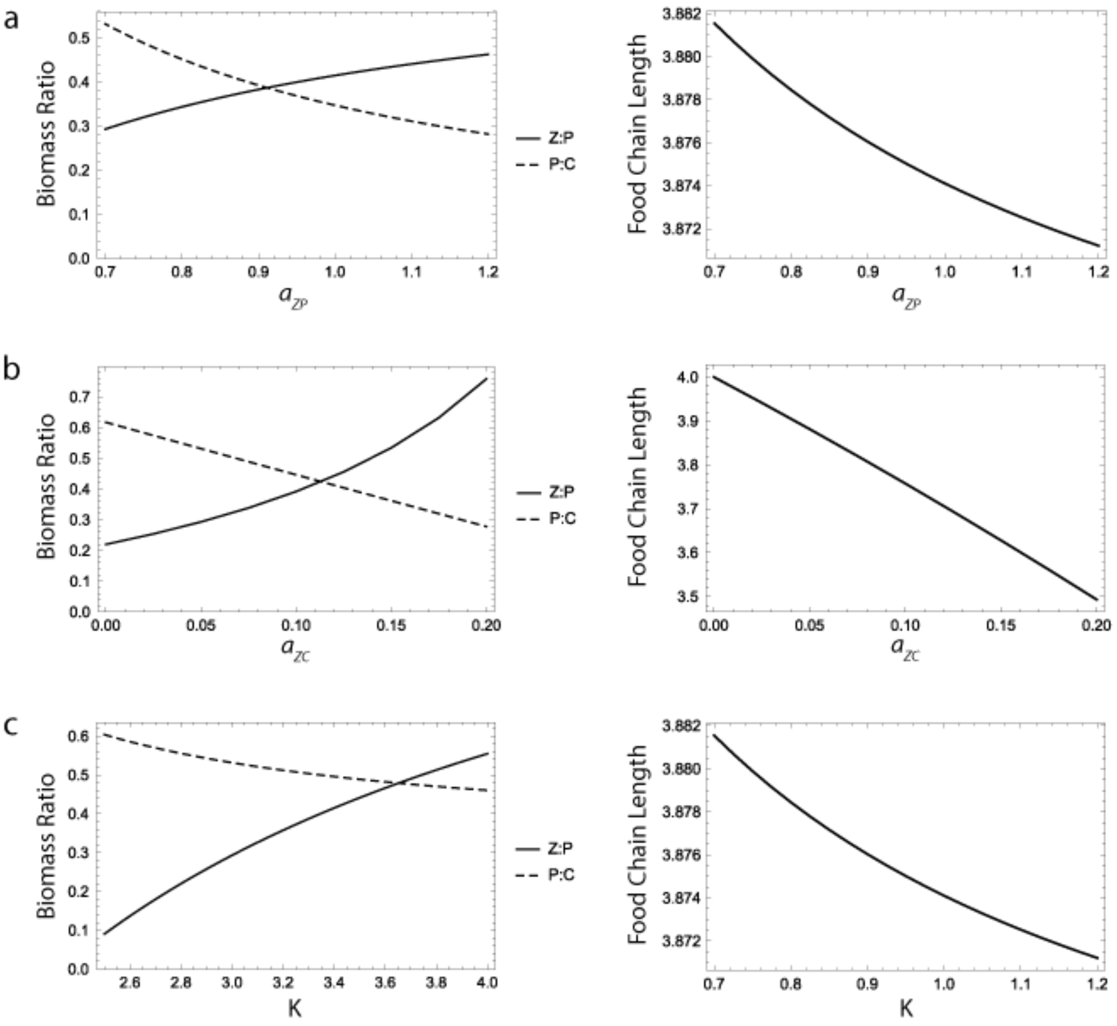

Supplementary Figure 2. Numerical solutions for Eqn. S1 for the effect of increasing (a) a_{ZP}, (b) a_{PC}, and (c) K on biomass pyramid shape and food chain length. Parameters : r = 2.0, K = 3.0, a_{CR} = 1.1, a_{PC} = 0.7, a_{ZP} = 0.7, a_{ZC} = 0.05, e = 1.0, m_C = 0.7, m_P= 0.7, m_Z = 0.5.

(b) Parallel food chains coupled by an omnivorous predator (Supplementary Fig. 1b):

$dR_1/dt = rR_1(1\text{-}R_1/K_1) - a_{C1R1}\, C_1R_1$

$dR_2/dt = rR_2(1\text{-}R_2/K_2) - a_{C2R2}\, C_2R_2$

$dC_1/dt = e_{C1R1}\ a_{C1R1}\, C_1R_1 - m_{C1}\, C_1 - a_{PC1}\, PC_1$ (S2)

$dC_2/dt = e_{C2R2}\ a_{C2R2}\, C_2R_2 - m_{C2}\, C_2 - a_{PC2}\, PC_2$

$dP/dt = e_{PC1}\, a_{PC1}\, PC_1 + e_{PR1}\ a_{PR1}\, PR_1 + e_{PC2}\, a_{PC2}\, PC_2 + e_{PR2}\ a_{PR2}\, PR_2 - m_P\, P$

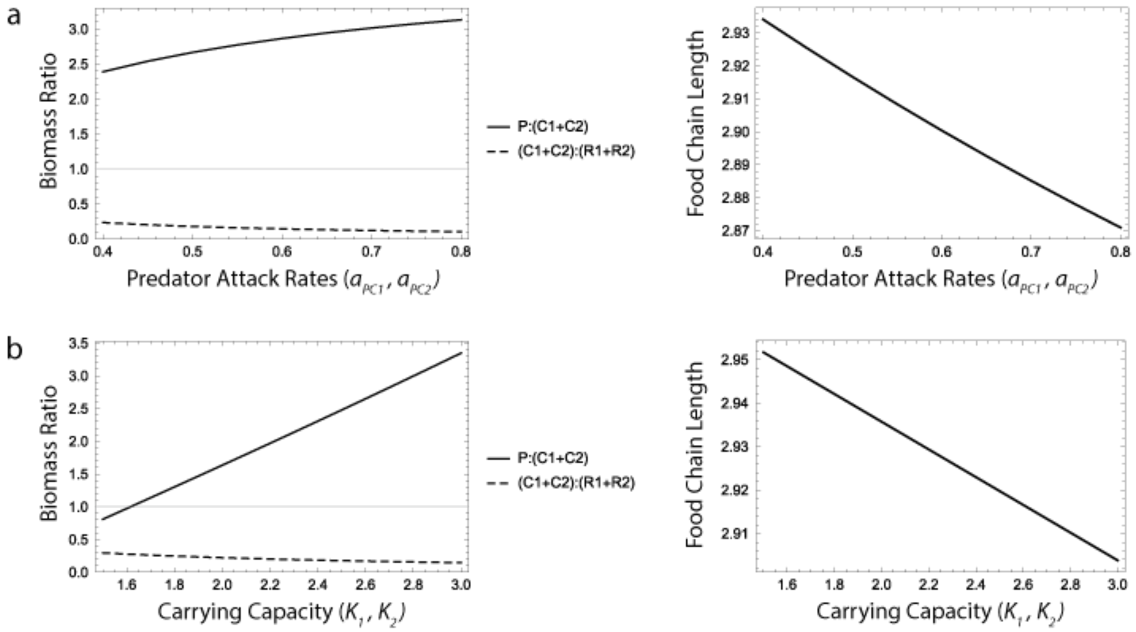

Supplementary Figure 3. Numerical solutions for Eqn. S2 for the effect of increasing (a) predator attack rates (a_{PC1}, a_{PC2}) and (b) carrying capacity (K_1, K_2) on biomass pyramid shape and food chain length. Parameters: $r_1 = 2.0$, $r_2 = 1.5$, $K_1 = 2.5$, $K_2 = 3.0$, $a_{C1R1} = 1.1$, $a_{C2R2} = 0.7$, $a_{PC1} = 0.7$, $a_{PC2} = 0.5$, $a_{PR1} = 0.01$, $a_{PR2} = 0.01$, $e = 1.0$, $m_{C1} = 0.7$, $m_{C2} = 0.7$, $m_P = 0.5$.

(c) Tri-trophic food chain with Type II functional responses:

$$dR/dt = rR(1\text{-}R/K) - a_{CR}\, CR\,/\,(R + b_{CR}) - a_{PR}\, PR\,/\,(R + b_{PR})$$

$$dC/dt = e_{CR}\; a_{CR}\, CR\,/\,(R + b_{CR}) - a_{PC}\, PC\,/\,(C + b_{PC}) - m_C\, C \qquad (S3)$$

$$dP/dt = e_{PC}\; a_{PC}\, PC\,/\,(C + b_{PC}) + e_{PR}\; a_{PR}\, PR\,/\,(R + b_{PR}) - m_P\, P$$

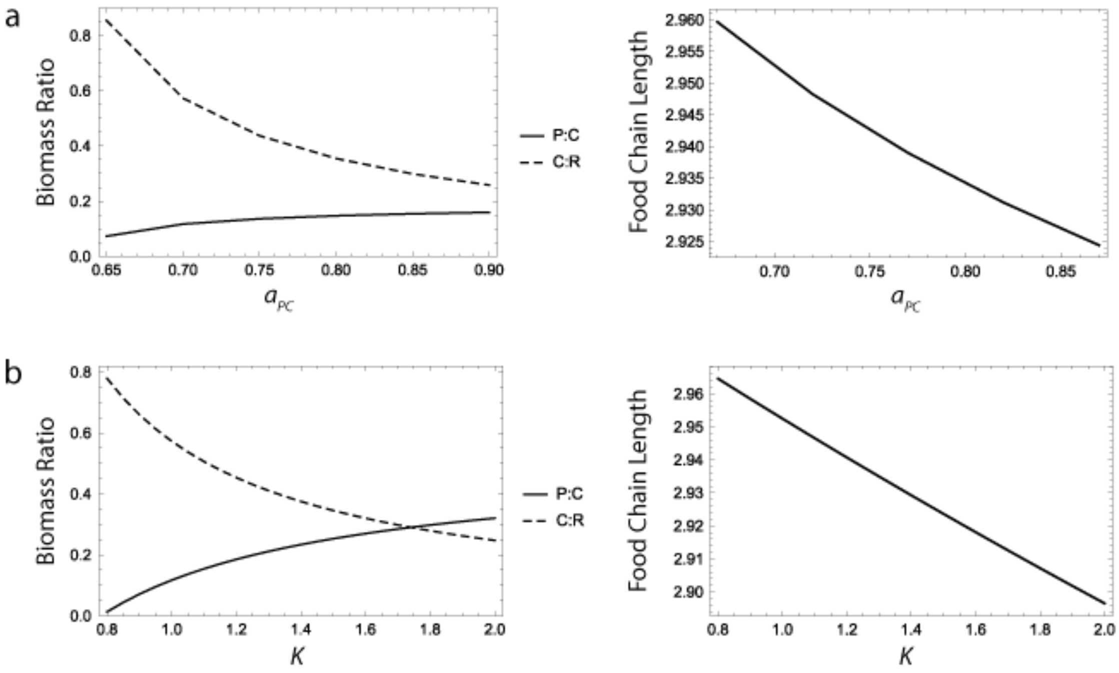

Supplementary Figure 4. Numerical solutions for Eqn. S3 for the effect of increasing (a) a_{PC} and (b) K on biomass pyramid shape and food chain length. Parameters: r = 2.0, K = 1.0, a_{CR} = 0.95, a_{PR} = 0.02, a_{PC} = 0.7, b_{CR} = 0.2, b_{PC} = 0.2, b_{PR} = 0.2, e = 1.0, m_C = 0.7, m_P = 0.5.

Supplementary Note 2
Evaluating support for the additive species richness mechanism for food chain length in lakes

In the absence of species richness estimates at the whole-community level, we were unable to evaluate the Classical Species Richness mechanism (CSRM; addition and insertion mechanisms) for food chain length (FCL). However, because top predators were uniquely fish in our lake dataset, estimates of fish species richness permitted evaluation of the addition / subtraction component of the CSRM.

Estimates of fish species richness for our lake dataset were derived from food web source publications or from literature whose stated purpose included the enumeration of fish species richness. When possible, estimates were cross-referenced using several sources, and where discrepancies existed we used the largest estimate found. Although we cannot assess the quality of these data using species accumulation curves[1], the data show the expected positive relationship with ecosystem size (log Fish Species Richness ~ log Ecosystem Size; $p <<< 0.001$, $R^2 = 0.57$, F-value = 42.16, n = 34). Moreover, our predictions involve qualitative patterns evaluated at a logarithmic scale, rendering results less susceptible to moderate inaccuracies in species richness. We evaluated the relationship between FCL and fish species richness using a linear model. We evaluated the relationship in lakes only because we found few robust estimates of fish species richness for our marine bounded systems.

FCL was not related to fish species richness in lake ecosystems when data were pooled across all levels of productivity and ecosystem size, nor among eutrophic (p = 0.38, n = 12), small (p = 0.16, n = 10), or large (p = 0.35, n = 24) lakes (Supplementary Fig. 5). Among oligo- and mesotrophic lakes, where our Energy Flux theory predicts that

FCL should be driven by ecosystem size, FCL was positively related to fish species richness (p = 0.036, R^2 = 0.203, F-value = 5.082, n = 22). Any positive relationship between ecosystem size and FCL may be driven by the Classical Species Richness or Energy Flux mechanisms. This correlation was likely due to the positive effect of ecosystem size on species richness (p <<< 0.001, R^2 = 0.70, F-value = 48, n = 22 for oligo- and mesotrophic lakes), rather than an effect of richness per se. This is supported by AICc values, which indicated that, among these 22 oligo- and mesotrophic lakes for which fish species richness data were available, a linear model for ecosystem size (FCL ~ log Ecosystem Size) fit the data better than a linear model for species richness (FCL ~ log Fish Species Richness; ΔAICc = 3.13). Moreover, a likelihood ratio test demonstrated that, among these lakes, a linear model with species richness and ecosystem size (FCL ~ log Fish Species Richness + log Ecosystem Size) did not fit the data better than a linear model with only log Ecosystem Size (D-statistic = 0.029, p = 0.86), but did provide a marginally better fit than a model with only log Fish Species Richness (D-statistic = 3.16, p = 0.075) – in other words, adding fish species richness did not improve the fit of a model which already included ecosystem size. Nevertheless, we cannot robustly distinguish between effects of the Additive Species Richness and Energy Flux mechanisms in low-productivity ecosystems.

These results suggest that the species addition component of the species richness mechanism was not supported among our lake data, although we cannot robustly distinguish between effects of the species addition and energy flux mechanisms in low-productivity ecosystems. We note several caveats to this analysis: (i) Fish data may reflect not only the additive species richness mechanism, but also the species insertion

mechanism, which may obscure the signal of the additive mechanism. (ii) Theory[2] and empirical work in natural settings suggest that species richness alone is often necessary yet insufficient to predict FCL, and instead acts in concert with other drivers of FCL (e.g. Supplementary Refs. 3 and 4). These observations suggest that FCL should not necessarily bear a strong relationship to species richness. (iii) Additionally, we may have failed to find support for the additive species richness mechanism because we use data from ecosystems with different regional species pools. Key to understanding the effect on our results is whether local environmental conditions (here, resource availability and ecosystem size) are more or less important than regional species richness in setting local species richness. We argue that assuming the greater importance of local saturation, as we do, is necessary for any such empirical test in natural settings regardless of whether the regional species pool is maintained constant – as such, our results bear similar bias to work conducted within a single regional species pool. Moreover, a regional pool effect should be less important in large and highly productive ecosystems (which should have species richness most resembling regional richness), and more important in small and oligotrophic ecosystems (which may or may not be locally saturated). As such, if local conditions are unimportant, there should be no relationship between ecosystem size and species richness, nor productivity and species richness – however, we document positive relationships between ecosystem size and fish species richness, and productivity and species richness in low-productivity ecosystems.

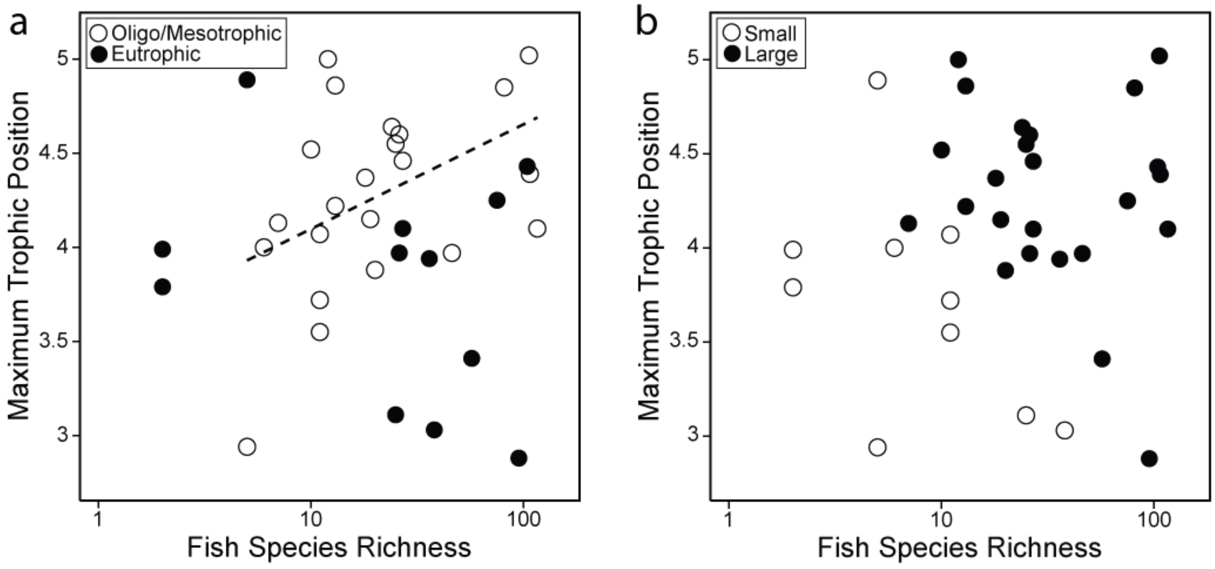

Supplementary Figure 5. The relationship between food chain length and fish species richness in lake ecosystems, among (a) oligo- and mesotrophic (dashed line) and eutrophic lakes, and among (b) small and large lakes.

Supplementary References

1. Gotelli, N.J. & Colwell, R.K. Quantifying biodiversity: procedures and pitfalls in the measurement and comparison of species richness. *Ecol. Lett.* **4,** 379-391 (2001).

2. Takimoto, G., Post, D.M., Spiller, D.A. & Holt, R.D. Effects of productivity, disturbance, and ecosystem size on food-chain length: insights from a metacommunity model of intraguild predation. *Ecol. Res.* **27,** 481-493 (2012).

3. McHugh, P.A., McIntosh, A.R. & Jellyman, P.G. Dual influences of ecosystem size and disturbance on food chain length in streams. *Ecol. Lett.* **13**, 881-890 (2010).

4. Young, H.S., et al. The roles of productivity and ecosystem size in determining food chain length in tropical terrestrial ecosystems. *Ecology*, **94**, 692–701(2013).
