# Supplementary material for: A mechanistic theory for aquatic food chain length
Source: Nat Commun. 2017 Dec 11;8:2028. doi: 10.1038/s41467-017-02157-0 (PMC5725575; doi:10.1038/s41467-017-02157-0)
Supplement: Supplementary file 3 — Description of Additional Supplementary Files [file 41467_2017_2157_MOESM3_ESM.pdf]

Description of Additional Supplementary Files

File Name: Supplementary Data 1
Description: Data for lake ecosystems. Original list of lakes and maximum trophic position is from Vander Zanden & Fetzer 2007 (Reference 22).

File Name: Supplementary Data 2
Description: Marine bounded ecosystems used in this study (from dataset compiled by Ward et al. 2015, ref. 13).
